# A Necessary and Sufficient Criterion for the Separability of Quantum State

**DOI:** 10.1038/s41598-018-19709-z

**Published:** 2018-01-23

**Authors:** Jun-Li Li, Cong-Feng Qiao

**Affiliations:** 1 University of the Chinese Academy of Sciences, Department of Physics, Beijing, 100049 China; 2 York University, Department of Physics & Astronomy, Toronto, ON M3J 1P3 Canada; 3 University of Chinese Academy of Sciences, Key Laboratory of Vacuum Physics, Beijing, 100049 China

## Abstract

Quantum entanglement has been regarded as one of the key physical resources in quantum information sciences. However, the determination of whether a mixed state is entangled or not is generally a hard issue, even for the bipartite system. In this work we propose an operational necessary and sufficient criterion for the separability of an arbitrary bipartite mixed state, by virtue of the multiplicative Horn’s problem. The work follows the work initiated by Horodecki *et al*. and uses the Bloch vector representation introduced to the separability problem by J. De Vicente. In our criterion, a complete and finite set of inequalities to determine the separability of compound system is obtained, which may be viewed as trade-off relations between the quantumness of subsystems. We apply the obtained result to explicit examples, e.g. the separable decomposition of arbitrary dimension Werner state and isotropic state.

## Introduction

Entanglement is a ubiquitous feature of quantum system and key element in quantum information processing, whereas yet far from fully understood^1^. A fundamental problem in the study of entanglement is the determination of the separability of quantum states. For pure state, the entangled states are those that cannot be expressed as the product of the subsystems, e.g. we say a bipartite pure state of *A* and *B* is entangled if it cannot be expressed in the product form like

1 $$|\psi {\rangle }_{AB}=|\varphi {\rangle }_{A}\otimes |\phi {\rangle }_{B}.$$

For the mixed state of a compound system, we say it is entangled if it cannot be written as a convex combination of product states. For example, a bipartite mixed state is separable (i.e. classically correlated^2^) whenever it can be expressed as

2 $${\rho }_{AB}=\sum _{i=1}^{L}\,{p}_{i}{\rho }_{i}^{(A)}\otimes {\rho }_{i}^{(B)}.$$

Here, ${\rho }_{i}^{(A,B)}$ are local density matrices of particles *A* and *B*; *p*_*i*_ > 0 and ${\sum }_{i=1}^{L}\,{p}_{i}=1$. The entanglement (non-separability) criterion for pure state is clear, by virtue of Schmidt or high order singular value decomposition for any-number-partite system^3^. However, none of the existing criteria for the separability of finite dimensional mixed states are satisfactory by far. They are generally either sufficient and necessary, but not practically usable; or easy to use, but only necessary (or only sufficient)^4^.

Over the past decades, one remarkable progress towards the operational characterization of a separable mixed state, the positive partial transposition (PPT) criterion^5^, was achieved by Peres twenty years ago. This separability criterion is only necessary and sufficient for 2 × 2 and 2 × 3 systems, rather than for arbitrary higher dimensional systems^6^. Though couple of necessary and sufficient criteria were developed afterwards^6–8^, they are generally difficult to handle in practice, or only applicable to low-rank density matrices^9^. With the dimension growing, the separability problem of a compound system tends to be NP-hard, even in the bipartite situation^10^. Recent investigations mostly focus on the necessary or sufficient conditions of witnessing entanglement or separability. The computable cross-norm or realignment (CCNR) criterion^11,12^ and local uncertainty relations (LURs)^13^ are proposed to detect entanglement. By virtue of the Bloch representation, separability criterion had been successfully formulated in matrix norms, which was found to be related to the CCNR criterion^14^. The optimization of entanglement witness observables may stand as a separability criterion^15^. For recent development, readers may refer to refs^16–18^ and more comprehensive reviews^19,20^. It should be noted that even restricted to necessary or sufficient criterion, the corresponding inequalities tend to be an ever growing family. Therefore, to find a complete and finite set of inequalities to determine the separability of mixed states is theoretically important and practically necessary.

In this work, we present an applicable criterion for the separability of bipartite mixed state through exploring the multiplicative Horn’s problem^21^. By expressing the quantum state in Bloch representation, the problem of factorizing a mixed state into sum of product states is transformed to the task of decomposing a matrix into the product of two other matrices. We find that the solution to the multiplicative Horn’s problem yields a complete and finite set of inequalities, a new criterion, which in practice provides a systematic procedure for the decomposition of separable mixed states. Relations between this new criterion and other related ones are investigated through concrete examples, including the separable decomposition of arbitrary dimensional Werner and isotropic states. Results manifest that the criterion raised in this work is to our knowledge the most applicable one at present in determining the separability of entangled quantum states.

## Results

### The Bloch representation of quantum state

A quantum state in *N*-dimensional Hilbert space may be expressed as^22^

3 $$\rho =\frac{1}{N}\mathbb{1}+\frac{1}{2}\,\sum _{\mu =1}^{{N}^{2}-1}\,{r}_{\mu }{\lambda }_{\mu },$$

where the real coefficients *r*_*μ*_ = 〈*λ*_*μ*_〉 = Tr[*ρλ*_*μ*_], and *λ*_*μ*_ are the *N*^2^ − 1 generators of SU(*N*) group. The *N*^2^ − 1 dimensional vector $\overrightarrow{r}={\{{r}_{1},\ldots ,{r}_{{N}^{2}-1}\}}^{{\rm{T}}}$ is called Bloch vector (or coherent vector) of the density matrix *ρ*, where the superscript T means the transposition. As the density matrix must be positive semidefinite and normalized, the vector $\overrightarrow{r}$ subjects to a set of constraints^23,24^, among which $|\overrightarrow{r}|\le \sqrt{\mathrm{2(}N-\mathrm{1)/}N}$ is imposed by the condition Tr[*ρ*^2^] ≤ 1 with the vector norm defined as $|\overrightarrow{r}|\equiv \sqrt{\overrightarrow{r}\cdot \overrightarrow{r}}$. Similarly, any bipartite state of dimensions *N* × *M* in the Bloch representation can be expressed as

4 $${\rho }_{AB}=\frac{1}{NM}\mathbb{1}\otimes \mathbb{1}+\frac{1}{2M}\overrightarrow{a}\cdot \overrightarrow{\lambda }\otimes \mathbb{1}+\frac{1}{2N}\mathbb{1}\otimes \overrightarrow{b}\cdot \overrightarrow{{\rm{\sigma }}}+\frac{1}{4}\,\sum _{\mu =1}^{{N}^{2}-1}\,\sum _{{\rm{\nu }}=1}^{{M}^{2}-1}\,{{\mathscr{T}}}_{\mu {\rm{\nu }}}{\lambda }_{\mu }\otimes {\sigma }_{{\rm{\nu }}}.$$

Here ${a}_{\mu }={\rm{Tr}}[{\rho }_{AB}({\lambda }_{\mu }\otimes \mathbb{1})]$, ${b}_{{\rm{\nu }}}={\rm{Tr}}[{\rho }_{AB}(\mathbb{1}\otimes {\sigma }_{{\rm{\nu }}})]$, ${{\mathscr{T}}}_{\mu {\rm{\nu }}}={\rm{Tr}}[{\rho }_{AB}({\lambda }_{\mu }\otimes {\sigma }_{{\rm{\nu }}})]$, and *σ*_ν_ are generators of SU(*M*). Reformulating the right hand side of Eq. (2) in term of Bloch representation of ${\rho }_{i}^{(A)}=\frac{1}{N}\mathbb{1}+\frac{1}{2}{\overrightarrow{r}}_{i}\cdot \overrightarrow{\lambda }$, ${\rho }_{j}^{(B)}=\frac{1}{M}\mathbb{1}+\frac{1}{2}{\overrightarrow{s}}_{j}\cdot \overrightarrow{\sigma }$, and comparing with Eq. (4), the necessary and sufficient condition of separability then turns to

5 $$\sum _{i}\,{p}_{i}{\overrightarrow{r}}_{i}=\overrightarrow{a},\,\sum _{j}\,{p}_{j}{\overrightarrow{s}}_{j}=\overrightarrow{b},\,\sum _{k=1}^{n}\,{p}_{k}{\overrightarrow{r}}_{k}\,{\overrightarrow{s}}_{k}^{{\rm{T}}}={\mathscr{T}},$$

where the subscripts in ${\overrightarrow{r}}_{i}$, ${\overrightarrow{s}}_{j}$ label different Bloch vectors rather the components of them, and the correlation matrix ${\mathscr{T}}$ has the matrix elements of ${{\mathscr{T}}}_{\mu {\rm{\nu }}}$.

The reduced density matrices of *A* and *B* can be derived from Eq. (4) by partial trace

6 $${\rho }_{A}={{\rm{Tr}}}_{B}[{\rho }_{AB}]=\frac{1}{N}\mathbb{1}+\frac{1}{2}\overrightarrow{a}\cdot \overrightarrow{\lambda },\,{\rho }_{B}={{\rm{Tr}}}_{A}[{\rho }_{AB}]=\frac{1}{M}\mathbb{1}+\frac{1}{2}\overrightarrow{b}\cdot \overrightarrow{{\rm{\sigma }}}.$$

Here the local ranks are rank(*ρ*_*A*_) = *n* and rank(*ρ*_*B*_) = *m*, where *n* and *m* may be non-full local ranks of *n* < *N* and *m* < *M*. We then have the following observation (see^25^ for details):

#### **Observation 1**.

*All N* × *M mixed states with local ranks n* < *N and m* < *M are either reducible to n* × *m states with full local ranks or entangled*.

According to Observation 1, we need only consider the separability of mixed states with full local ranks. The full local rank state could be transformed to a normal form with maximally mixed subsystems^26^, i.e.

7 $${\rho }_{AB}\to {\tilde{\rho }}_{AB}=\frac{1}{NM}\mathbb{1}+\frac{1}{4}\,\sum _{\mu ,{\rm{\nu }}=1}^{{N}^{2}-\mathrm{1,}{M}^{2}-1}\,{\tilde{{\mathscr{T}}}}_{\mu {\rm{\nu }}}{\lambda }_{\mu }\otimes {{\rm{\sigma }}}_{{\rm{\nu }}}.$$

Note that in the literature there are studies about the normal form in the separability problem^27,28^. Hereafter, the quantum states *ρ*_*AB*_ are implied to be in their normal form, and we have

#### **Observation 2**.

*Let* ${\overrightarrow{r}}_{i}$ *and* ${\overrightarrow{s}}_{j}$ *be Bloch vectors of density matrices and* $\overrightarrow{p}={({p}_{1},{p}_{2},\ldots ,{p}_{L})}^{{\rm{T}}}$, *we may define two matrices* ${M}_{rp}\equiv {M}_{r}{D}_{p}^{\frac{1}{2}}$ *and* ${M}_{sp}\equiv {M}_{s}{D}_{p}^{\frac{1}{2}}$, *where* ${M}_{r}=\{{\overrightarrow{r}}_{1},{\overrightarrow{r}}_{2},\ldots ,{\overrightarrow{r}}_{L}\}$, ${M}_{s}=\{{\overrightarrow{s}}_{1},{\overrightarrow{s}}_{2},\ldots ,{\overrightarrow{s}}_{L}\}$, *and D*_*p*_ = diag{*p*_1_, *p*_2_, …, *p*_*L*_} *with* 0 < *p*_*i*_ ≤ 1 *and* ${\sum }_{i=1}^{L}\,{p}_{i}=1$. *The state ρ*_*AB*_*is separable if and only if there exist a number L such that* ${\mathscr{T}}={M}_{rp}{M}_{sp}^{{\rm{T}}}$ *with* ${M}_{r}\overrightarrow{p}=0$ *and* ${M}_{s}\overrightarrow{p}=0$.

### The criterion of separability

For arbitrary bipartite quantum state *ρ*_*AB*_ in normal form, let ${\mathscr{T}}$ be its correlation matrix, and *M*_*rp*_ and *M*_*sp*_ be defined in Observation 2, the decomposition ${\mathscr{T}}={M}_{rp}{M}_{sp}^{{\rm{T}}}$ then can be obtained via the following theorem:

#### **Theorem 1**.

*A real matrix* ${\mathscr{T}}$ *can be decomposed as* ${\mathscr{T}}={M}_{rp}{M}_{sp}^{{\rm{T}}}$ *if and only if M*_*rp*_, *M*_*sp*_, *and D*_*τ*_*have the following relation*

8 $${M}_{rp}=({\overrightarrow{u}}_{1},\ldots ,{\overrightarrow{u}}_{L})X{D}_{\alpha }{Q}^{\mathrm{(1)}},$$

9 $${M}_{sp}=({\overrightarrow{v}}_{1},\ldots ,{\overrightarrow{v}}_{L})Y{D}_{\beta }{Q}^{\mathrm{(2)}},$$

10 $${D}_{\tau }=X{D}_{\alpha }{Q}^{\mathrm{(1)}}{Q}^{\mathrm{(2)}{\rm{T}}}{D}_{\beta }{Y}^{{\rm{T}}}.$$

*Here*, ${\overrightarrow{u}}_{i}$ *and* ${\overrightarrow{v}}_{j}$ *are the left and right singular vectors of* ${\mathscr{T}}={\sum }_{i=1}^{L}\,{\tau }_{i}{\overrightarrow{u}}_{i}{\overrightarrow{v}}_{i}^{{\rm{T}}}$ *with singular values of τ*_*i*_; *X*, *Y*, *Q*^(1)^, *Q*^(2)^ ∈ SO(*L*); *D*_*α*_ = diag{*α*_1_, …, *α*_*L*_} *and D*_*β*_ = diag{*β*_1_, …, *β*_*L*_} *are singular values of M*_*rp*_*and M*_*sp*_; *D*_*τ*_ = diag{*τ*_1_, …, *τ*_*L*_}, *with* $L > {\rm{rank}}({\mathscr{T}}\,)=l$; *all the singular values are arranged in descending order*.

#### **Proof:**

The if part is quite streightfoward

11 $${M}_{rp}{M}_{sp}^{{\rm{T}}}=({\overrightarrow{u}}_{1},\ldots ,{\overrightarrow{u}}_{L})X{D}_{\alpha }{Q}^{(1)}{Q}^{(2){\rm{T}}}{D}_{\beta }{Y}^{{\rm{T}}}(\begin{array}{c}{\overrightarrow{\nu }}_{1}^{{\rm{T}}}\\ \vdots \\ {\overrightarrow{\nu }}_{L}^{{\rm{T}}}\end{array})=({\overrightarrow{u}}_{1},\ldots ,{\overrightarrow{u}}_{L}){D}_{\tau }(\begin{array}{c}{\overrightarrow{\nu }}_{1}^{{\rm{T}}}\\ \vdots \\ {\overrightarrow{\nu }}_{L}^{{\rm{T}}}\end{array})={\mathscr{T}}.$$

For the only if part, suppose the singular value decompositions of *M*_*rp*_ and *M*_*sp*_ are

12 $${M}_{rp}=({\overrightarrow{u}^{\prime} }_{1},\ldots ,{\overrightarrow{u}^{\prime} }_{L}){D}_{\alpha }Q,\,{M}_{sp}=({\overrightarrow{{\rm{\nu }}}^{\prime} }_{1},\ldots ,{\overrightarrow{{\rm{\nu }}}^{\prime} }_{L}){D}_{\beta }Q^{\prime} ,$$

we have

13 $${M}_{rp}{M}_{sp}^{{\rm{T}}}=({\overrightarrow{u{\rm{^{\prime} }}}}_{1},\ldots ,{\overrightarrow{u{\rm{^{\prime} }}}}_{L}){D}_{\alpha }Q{\overrightarrow{Q{\rm{^{\prime} }}}}^{{\rm{T}}}{D}_{\beta }(\begin{array}{c}{\overrightarrow{v{\rm{^{\prime} }}}}_{1}^{{\rm{T}}}\\ \vdots \\ {\overrightarrow{v{\rm{^{\prime} }}}}_{L}^{{\rm{T}}}\end{array}).$$

Because ${\mathscr{T}}={M}_{rp}{M}_{sp}^{{\rm{T}}}$, the singular value decomposition of the matrix *D*_*α*_*QQ*′^T^*D*_*β*_ must be *D*_*α*_*QQ*′^T^*D*_*β*_ = *X*^T^*D*_*τ*_*Y*, where *D*_*τ*_ contains the first *L* singular values of ${\mathscr{T}}$. Therefore, we have

14 $${M}_{rp}{M}_{sp}^{{\rm{T}}}=({\overrightarrow{u{\rm{^{\prime} }}}}_{1},\ldots ,{\overrightarrow{u{\rm{^{\prime} }}}}_{L}){X}^{{\rm{T}}}{D}_{\tau }Y(\begin{array}{c}{\overrightarrow{v{\rm{^{\prime} }}}}_{1}^{{\rm{T}}}\\ \vdots \\ {\overrightarrow{v{\rm{^{\prime} }}}}_{L}^{{\rm{T}}}\end{array})$$

with $$({\overrightarrow{u}^{\prime} }_{1},\ldots ,{\overrightarrow{u}^{\prime} }_{L}){X}^{{\rm{T}}}=({\overrightarrow{u}}_{1},\ldots ,{\overrightarrow{u}}_{L})$$ and $$({\overrightarrow{v}^{\prime} }_{1},\ldots ,{\overrightarrow{v}^{\prime} }_{L}){Y}^{{\rm{T}}}=({\overrightarrow{v}}_{1},\ldots ,{\overrightarrow{v}}_{L})$$. Q.E.D.

Observation 2 turns the separable problem of a compound system to the question of how to decompose the correlation matrix into a product of other two nontrivial matrices, i.e. ${\mathscr{T}}={M}_{rp}{M}_{sp}^{{\rm{T}}}$, with constraints ${M}_{r}\overrightarrow{p}=0$ and ${M}_{s}\overrightarrow{p}=0$. Theorem 1 further gives the decomposition conditions, that is: (1) The left singular vectors of *M*_*rp*_ and *M*_*sp*_ agree with the left and right singular vectors of ${\mathscr{T}}$ (i.e. Eqs (8 and 9)); (2) The right singular vectors of *M*_*rp*_ and *M*_*sp*_, and their singular values satisfy Eq. (10). For condition 1, we may rotate the orthogonal bases of particle *A* and *B* to $\{{\overrightarrow{u}}_{i}\}$ and $\{{\overrightarrow{v}}_{i}\}$ respectively, where ${\mathscr{T}}$ becomes a diagonal matrix. While for condition 2, we need to solve Eq. (10), which makes the singular values of matrices ${\mathscr{T}}$, *M*_*rp*_, and *M*_*sp*_ correlated.

Before proceeding to the Eq. (10), two prerequisite lemmas are necessary. Let *I*, *J*, *K* be certain subsets of natural numbers {1, …, *n*} with the same cardinality *r*, i.e., *I* = {*i*_1_, *i*_2_, …, *i*_*r*_}, *J* = {*j*_1_, *j*_2_, …, *j*_*r*_}, and *K* = {*k*_1_, *k*_2_, …, *k*_*r*_}, where the elements are arranged in increasing order so that ${i}_{r} > {i}_{r-1} > \cdots  > {i}_{1}$; ${j}_{r} > {j}_{r-1} > \cdots  > {j}_{1}$, and ${k}_{r} > {k}_{r-1} > \cdots  > {k}_{1}$. Define ${\mathcal F} (I)\equiv ({i}_{r}-r,{i}_{r-1}-(r-\mathrm{1),}\ldots ,{i}_{1}-\mathrm{1)}$, and let the triplet $(\lambda ,\mu ,{\rm{\nu }})=$ $( {\mathcal F} (I), {\mathcal F} (J), {\mathcal F} (K))$, then we are legitimate to introduce a triple set ${T}_{r}^{n}=\{(I,J,K)\}$ defined as:

#### **Lemma 1**.

*A triplet* (*I*, *J*, *K*) *is in* ${T}_{r}^{n}$ *if and only if the corresponding triplet* (*λ*, *μ*, ν) *occurs as eigenvalues of the triple of r by r Hermitian matrices*, *with the third to be the sum of the first two*.

This lemma appears as Theorem 2 of ref.^29^, where the practical methods on how to generate ${T}_{r}^{n}$ were also discussed, i.e., via the Horn’s inductive procedure or Littlewood-Richardson coefficients.

#### **Lemma 2**.

*A triplet* ({*a*_*i*_}, {*b*_*i*_}, {*c*_*i*_}) *occurs as singular values of n by n real matrices A*, *B*, *and C*(*C* = *AB*) *if and only if*

15 $$\prod _{k\in K}\,{c}_{k}\le \prod _{i\in I}\,{a}_{i}\cdot \prod _{j\in J}\,{b}_{j}$$

*for all* (*I*, *J*, *K*) *in* ${T}_{r}^{n}$ *and all r* < *n*.

This is known as the multiplicative Horn’s problem, see theorem 16 of ref.^29^ for details. Historically, the multiplicative Horm’s problem first appeared as the Thompson’s conjecture^30^, and later was found can be solved for invertible matrices^31^. It was found to be true for real matrices^32^, and even extendable to the case of non-invertible matrices recently^21^ (see Supplemental Material for a brief review of the proof).

The decomposition of Eq. (10) can be realized through the following theorem:

#### **Theorem 2**.

*There exists a real orthogonal matrix Q such that D*_*α*_*QD*_*β*_*has the singular values of D*_*τ*_, *if and only if the following is satisfied*

16 $$\prod _{k\in K}\,{\tau }_{k}\le \prod _{i\in I}\,{\alpha }_{i}\,\prod _{j\in J}\,{\beta }_{j},$$

*for all* $(I,J,K)\in {T}_{r}^{L}$ *and r* < *L*.

Theorem 2 is a direct application of Lemma 2. For a given bipartite state whose correlation matrix ${\mathscr{T}}$ is known, the Eq. (16) applies to all possible singular values of the matrices decomposed from ${\mathscr{T}}$, and behaves as trade-off relations among them. The singular values of *M*_*rp*_ and *M*_*sp*_ are determined by their column vectors, i.e. ${\overrightarrow{r}}_{i}$ and ${\overrightarrow{s}}_{i}$, whose norms relate to the mixedness (or quantumness) of the subsystems, i.e. ${\rho }_{i}^{(A)}$ and ${\rho }_{i}^{(B)}$. Large *τ*_*i*_ implies large *α*_*i*_ or *β*_*i*_ or both. When column vectors ${\overrightarrow{r}}_{i}$ and ${\overrightarrow{s}}_{i}$ surpass the Bloch vectors of density matrices in lengths, the quantum state ${\mathscr{T}}$ is entangled. The quantum state is separable only when the two factor matrices are composed of Bloch vectors of physical states.

In the following we demonstrate our method in bipartite quantum system as an example. For more systematic and detailed applications, readers may refer to ref.^25^. It should be noted that theorems 1 and 2 are also suitable to the bi-separability of arbitrary multipartite states, and hence the method presented here is also applicable to the multi-separability problem, due to the reason that the Bloch representation generally turns the sum decomposition problem into a product decomposition one.

### Applications

In Bloch representation of quantum state, we have the following two observations:

#### **Observation 3**.

*If* $\overrightarrow{r}$ *is a Bloch vector of a density matrix*, *then the* $\overrightarrow{r}^{\prime}$, *whose components* ${r^{\prime} }_{\mu }=-{r}_{\mu }$ *corresponding to those SU*(*N*) *generators satisfying* ${\lambda }_{\mu }^{{\rm{T}}}=-{\lambda }_{\mu }$, *is also a Bloch vector of a density matrix*.

#### **Observation 4**.

*If the norm of a Bloch vector with dimension* (*N*^2^ − 1) *satisfies* $|\overrightarrow{r}{|}^{2}\le \frac{2}{N(N-\mathrm{1)}}$, *then* ${\overrightarrow{r}}^{{\rm{^{\prime} }}}=P\overrightarrow{r}$ *is also a Bloch vector for arbitrary rotation P* ∈  *SO*(*N*^2^ − 1).

Here, the Observation 3 is established due to the fact that the transposition of a positive semidefinite Hermitian matrix keeps on being positive semidefinite, while the Observation 4 is just a corollary of Eq. (11) in ref.^14^ (or see ref.^33^). In the following, we demonstrate how the criterion works through concrete examples.

#### **Example I:**

*The relationship between Vicente*’*s criterion*^14^ *and ours*

A subset inequalities of Eq. (16) goes as follows (see theorem 3.3.4 of ref.^34^):

17 $$\prod _{i=1}^{k}\,{\tau }_{i}\le \prod _{i=1}^{k}\,{\alpha }_{i}{\beta }_{i},\,k=1,2,\ldots .$$

Employing Ky Fan matrix norm $\parallel {\mathscr{T}}{\parallel }_{{\rm{KF}}}={\sum }_{i=1}^{L}\,{\tau }_{i}$ and Schwarz inequality ${\sum }_{i}\,{\alpha }_{i}{\beta }_{i}\le {({\sum }_{i}{\alpha }_{i}^{2})}^{\mathrm{1/2}}{({\sum }_{i}{\beta }_{i}^{2})}^{\mathrm{1/2}}$, we have:

#### **Corollary 1**.

*The average square norms of the local states*’ *Bloch vectors are lower bounded by Ky Fan norm of the correlation matrix*

18 $$(\sum _{i=1}^{L}\,{p}_{i}|{\overrightarrow{r}}_{i}{|}^{2})\,(\sum _{j=1}^{L}\,{p}_{j}|{\overrightarrow{s}}_{j}{|}^{2})\ge \parallel {\mathscr{T}}\,{\parallel }_{{\rm{KF}}}^{2}.$$

#### Proof:

Eq. (17) leads to (see Corollary 3.3.10 of ref.^34^)

19 $$\sum _{i=1}^{k}\,{\tau }_{i}\le \sum _{i=1}^{k}\,{\alpha }_{i}{\beta }_{i},\,k\in \{1,\ldots ,L\}.$$

The Ky Fan norm of ${\mathscr{T}}$ is

20 $$\parallel {\mathscr{T}}\,{\parallel }_{{\rm{KF}}}=\sum _{i}\,{\tau }_{i}\le \sum _{i}\,{\alpha }_{i}{\beta }_{i}\le {(\sum _{i}{\alpha }_{i}^{2})}^{\frac{1}{2}}\,{(\sum _{i}{\beta }_{i}^{2})}^{\frac{1}{2}}.$$

The Frobennius norm for real matrices are $\parallel M{\parallel }_{2}\equiv {\rm{Tr}}{[{M}^{{\rm{T}}}M]}^{\frac{1}{2}}$, so we have

21 $$\sum _{i}\,{\alpha }_{i}^{2}={\rm{Tr}}[{M}_{rp\,}^{{\rm{T}}}{M}_{rp}]=\sum _{i}\,{p}_{i}|{\overrightarrow{r}}_{i}{|}^{2},\,\sum _{i}\,{\beta }_{i}^{2}={\rm{Tr}}[{M}_{sp\,}^{{\rm{T}}}{M}_{sp}]=\sum _{i}\,{p}_{i}|{\overrightarrow{s}}_{i}{|}^{2}.$$

Q.E.D.

Because $|{\overrightarrow{r}}_{i}{|}^{2}\le \frac{\mathrm{2(}N-\mathrm{1)}}{N}$ and $|{\overrightarrow{s}}_{i}{|}^{2}\le \frac{\mathrm{2(}M-\mathrm{1)}}{M}$, we have

22 $$\sum _{i=1}^{L}\,{p}_{i}|{\overrightarrow{r}}_{i}{|}^{2}\le \frac{\mathrm{2(}N-\mathrm{1)}}{N},\,\sum _{i=1}^{L}\,{p}_{i}|{\overrightarrow{s}}_{i}{|}^{2}\le \frac{\mathrm{2(}M-\mathrm{1)}}{M}.$$

Taking Eq. (22) into Corollary 1, Theorem 1 of ref.^14^ is arrived. On the other hand, from Observation 4 we have the following:

#### **Corollary 2**.

*If* $\parallel {\mathscr{T}}\,{\parallel }_{{\rm{KF}}}\le \frac{2}{\sqrt{MN(M-\mathrm{1)}\,(N-\mathrm{1)}}}$, *the quantum state* ${\mathscr{T}}$ *is separable*.

#### **Proof:**

Suppose that ${\mathscr{T}}={\sum }_{i=1}^{l}\,{\tau }_{i}{\overrightarrow{u}}_{i}\,{\overrightarrow{v}}_{i}^{{\rm{T}}}$ with rank(*T*) = *l* ≥ 1, when working in the bases of ${\overrightarrow{u}}_{i}$ and ${\overrightarrow{v}}_{i}$, we may construct the following matrix equation

23 $$(\begin{array}{ccccc}{\tau }_{1} & 0 & \cdots  & 0 & 0\\ 0 & {\tau }_{2} & \cdots  & 0 & 0\\ \vdots  & \vdots  & \ddots  & \vdots  & \vdots \\ 0 & 0 & \cdots  & {\tau }_{l} & 0\\ 0 & 0 & \cdots  & 0 & 0\end{array})=(\begin{array}{ccccc}{\alpha }_{1} & 0 & \cdots  & 0 & 0\\ 0 & {\alpha }_{2} & \cdots  & 0 & 0\\ \vdots  & \vdots  & \ddots  & \vdots  & \vdots \\ 0 & 0 & \cdots  & {\alpha }_{l} & 0\\ 0 & 0 & \cdots  & 0 & 0\end{array})\,Q{Q}^{{\rm{T}}}\,(\begin{array}{ccccc}{\beta }_{1} & 0 & \cdots  & 0 & 0\\ 0 & {\beta }_{2} & \cdots  & 0 & 0\\ \vdots  & \vdots  & \ddots  & \vdots  & \vdots \\ 0 & 0 & \cdots  & {\beta }_{l} & 0\\ 0 & 0 & \cdots  & 0 & 0\end{array}).$$

Here, *Q* ∈ SO(*l* + 1) with elements in the last row being ${Q}_{(l+\mathrm{1)}j}=\sqrt{{p}_{j}}$; *p*_*j*_ ≥ 0, and ${\sum }_{j=1}^{l+1}\,{p}_{j}=1$. Choosing ${\alpha }_{i}={(\frac{2}{N(N-\mathrm{1)}})}^{\frac{1}{2}}\sqrt{{\kappa }_{i}}$, ${\beta }_{i}={(\frac{2}{M(M-\mathrm{1)}})}^{\frac{1}{2}}\sqrt{{\kappa }_{i}}$, and ${\kappa }_{i}={\tau }_{i}\frac{\sqrt{N(N-\mathrm{1)}M(M-\mathrm{1)}}}{2}$, we have

24 $${\tau }_{i}={\alpha }_{i}{\beta }_{i},\,{\mathscr{K}}\equiv \sum _{i=1}^{l}\,{\kappa }_{i}=\frac{\sqrt{N(N-\mathrm{1)}M(M-\mathrm{1)}}}{2}\,\sum _{j=1}^{l}\,{\tau }_{i}\le 1.$$

Comparing Eq. (23) with ${\mathscr{T}}={M}_{rp}{M}_{sp}^{{\rm{T}}}$, we can get the Bloch vectors ${\overrightarrow{r}}_{j}$ and ${\overrightarrow{s}}_{j}$

25 $$\sqrt{{p}_{j}}\,{\overrightarrow{r}}_{j}={({\alpha }_{1}{Q}_{1j},{\alpha }_{2}{Q}_{2j},\ldots ,{\alpha }_{l}{Q}_{lj})}^{{\rm{T}}},$$

26 $$\sqrt{{p}_{j}}\,{\overrightarrow{s}}_{j}={({\beta }_{1}{Q}_{1j},{\beta }_{2}{Q}_{2i},\ldots ,{\beta }_{l}{Q}_{lj})}^{{\rm{T}}},$$

where *j* ∈ {1, …, *l* + 1} and the norms are

27 $${p}_{j}|{\overrightarrow{r}}_{j}{|}^{2}=\sum _{i=1}^{l}\,{\alpha }_{i}^{2}{Q}_{ij}^{2}=\frac{2}{N(N-\mathrm{1)}}\,\sum _{i=1}^{l}\,{\kappa }_{i}{Q}_{ij}^{2},$$

28 $${p}_{j}|{\overrightarrow{s}}_{j}{|}^{2}=\sum _{i=1}^{l}\,{\beta }_{i}{Q}_{ij}^{2}=\frac{2}{M(M-\mathrm{1)}}\,\sum _{i=1}^{l}\,{\kappa }_{i}{Q}_{ij}^{2}.$$

We may set the probability distribution *p*_*j*_ to be ${p}_{j}=\frac{1}{{\mathscr{K}}}\,{\sum }_{i=1}^{l}\,{\kappa }_{i}{Q}_{ij}^{2}$. Then replacing the *p*_*j*_ in Eqs (27, 28), we have

29 $$|{\overrightarrow{r}}_{j}{|}^{2}=\frac{2{\mathscr{K}}}{N(N-\mathrm{1)}}\le \frac{2}{N(N-\mathrm{1)}},\,|{\overrightarrow{s}}_{j}{|}^{2}=\frac{2{\mathscr{K}}}{M(M-\mathrm{1)}}\le \frac{2}{M(M-\mathrm{1)}}.$$

According to Observation 4, the Corollary 2 is then established. Q.E.D.

Corollary 2 agrees with the Proposition 3 of ref.^14^ where *M* and *N* are dimensions of the subsystems. Here, in proof of Corollary 2, explicit separable decomposition of ${\mathscr{T}}$ into *M*_*rp*_ and *M*_*sp*_ with ${M}_{r}\overrightarrow{p}={M}_{s}\overrightarrow{p}=0$ also exhibits.

#### **Example II:**

*The relation with PPT* ^5^ *scheme*

The partial transposition of a bipartite density matrix corresponds to the sign flips of columns or rows (not both) of ${\mathscr{T}}$, whose indices are that of skew symmetric generators, i.e., ${\lambda }_{\mu }^{{\rm{T}}}=-{\lambda }_{\mu }$. The Observation 3 implies that the PPT criterion is necessary for separability. Conversely, the positivity of partially transposed density matrix generally does not imply separability, that is, PPT is not sufficient. However, for qubit-qubit system, calculation indicates that the PPT of density operators gives $0\le {\sum }_{i}\,{\tau }_{i}\le 1$ by means of the technique introduced in ref.^35^ (see Supplemental Material). As $1\le \frac{2}{\mathrm{2(2}-\mathrm{1)}}$, according to the Corollary 2, PPT also tells separability. Therefore it is a necessary and sufficient condition for qubit-qubit system, which agrees with the conclusionn proved by other method^6^.

#### **Example III:**

*For generalized Werner state and isotropic state*

### The relation between the Werner state and the isotropic state

The generalized Werner state and isotropic state in the Bloch vector representation read^14^:

30 $${\rho }_{{\rm{W}}}=\frac{1}{{N}^{2}}\mathbb{1}\otimes \mathbb{1}+\frac{1}{4}\,\sum _{\mu =1}^{{N}^{2}-1}\,\frac{\mathrm{2(}N\varphi -\mathrm{1)}}{N({N}^{2}-\mathrm{1)}}{\lambda }_{\mu }\otimes {\lambda }_{\mu },$$

31 $${\rho }_{{\rm{ISO}}}=\frac{1}{{N}^{2}}\mathbb{1}\otimes \mathbb{1}+\frac{1}{4}\,(\sum _{\mu \in {S}_{1}}\,\frac{2p}{N}{\lambda }_{\mu }\otimes {\lambda }_{\mu }-\sum _{\nu \in {S}_{2}}\,\frac{2p}{N}{\lambda }_{\nu }\otimes {\lambda }_{\nu }),$$

where *S*_1_ represents the symmetric generators of ${\lambda }_{\mu }^{{\rm{T}}}={\lambda }_{\mu }$, and *S*_2_ denotes the skew symmetric generators of ${\lambda }_{\nu }^{{\rm{T}}}=-{\lambda }_{\nu }$. The partial transposition operation correlates the Werner state with isotropic states. According to Observation 3, we may readily find that the parameters in Eqs (30 and 31) satisfies

32 $$p=\frac{N\varphi -1}{{N}^{2}-1}.$$

Equation (32) tells us that, when considering the separability, only one of the two states need to be taken into account. Before proceeding to the separable decomposition, we first present serval straightforward but interesting results from Eq. (32): (1) The positivity condition $\frac{-\,1}{{N}^{2}-1}\le p$ of *ρ*_ISO_^36^ implies that *ρ*_W_ is entangled when *ϕ* < 0; (2) The positivity condition *ϕ* ≤ 1 of *ρ*_W_ implies that *ρ*_ISO_ is entangled when $\frac{1}{N+1} < p$; (3) If *ρ*_W_ is separable at 0 ≤ *ϕ* ≤ 1 then *ρ*_ISO_ is separable at $\frac{-\,1}{{N}^{2}-1}\le p\le \frac{1}{N+1}$.

### Separable decomposition of the Werner state

Considering the Werner state with ${\mathscr{T}}=\frac{\mathrm{2(}N\varphi -\mathrm{1)}}{N({N}^{2}-\mathrm{1)}}1$ and ${\rm{rank}}({\mathscr{T}})={N}^{2}-1$, there must be at least ${N}^{2}$ Bloch vectors in both *M*_*rp*_ and *M*_*sp*_, due to the additional constraints ${M}_{r}\overrightarrow{p}=0$ and ${M}_{s}\overrightarrow{p}=0$. Based on Theorem 2, we may construct *M*_*rp*_ and *M*_*sp*_ as follows:

33 $${M}_{rp}={M}_{r}{D}_{p}^{\frac{1}{2}}=(\begin{array}{ccccc}{\alpha }_{1} & 0 & \cdots  & 0 & 0\\ 0 & {\alpha }_{2} & \cdots  & 0 & 0\\ \vdots  & \vdots  & \ddots  & \vdots  & \vdots \\ 0 & 0 & \cdots  & {\alpha }_{{N}^{2}-1} & 0\end{array})\,Q,$$

34 $${M}_{sp}={M}_{s}{D}_{p}^{\frac{1}{2}}=(\begin{array}{ccccc}{\beta }_{1} & 0 & \cdots  & 0 & 0\\ 0 & {\beta }_{2} & \cdots  & 0 & 0\\ \vdots  & \vdots  & \ddots  & \vdots  & \vdots \\ 0 & 0 & \cdots  & {\beta }_{{N}^{2}-1} & 0\end{array})\,Q.$$

Here, ${D}_{p}={\rm{diag}}\{{p}_{1},\ldots ,{p}_{{N}^{2}}\}$; *Q* ∈ SO(*N*^2^) whose elements in last row are ${Q}_{{N}^{2}i}=\sqrt{{p}_{i}}$ which ensures${M}_{r}\overrightarrow{p}=$ ${M}_{s}\overrightarrow{p}=0$. Since the singular values of ${\mathscr{T}}$ are all equal, we may set ${\alpha }_{1}=\cdots ={\alpha }_{{N}^{2}-1}=\alpha$, ${\beta }_{1}=\cdots ={\beta }_{{N}^{2}-1}=\beta$, and ${p}_{1}=\cdots ={p}_{{N}^{2}}=\frac{1}{{N}^{2}}$; hence have

35 $$\frac{1}{N}{\overrightarrow{r}}_{i}=\alpha {({Q}_{1i},{Q}_{2i},\ldots ,{Q}_{({N}^{2}-\mathrm{1)}i})}^{{\rm{T}}},\,\frac{1}{N}{\overrightarrow{s}}_{i}=\beta {({Q}_{1i},{Q}_{2i},\ldots ,{Q}_{({N}^{2}-\mathrm{1)}i})}^{{\rm{T}}}.$$

The sets $\{{\overrightarrow{r}}_{i}|i=1,\ldots ,{N}^{2}\}$ and $\{{\overrightarrow{s}}_{i}|i=1,\ldots ,{N}^{2}\}$ form two *N*^2^-simplexes (or hypertetrahedron) each of which lies in the (*N* ^2^ − 1)-dimensional Bloch vector space of particles *A* and *B*. The angles between any two of the Bloch vectors fulfil

36 $$\frac{{\overrightarrow{r}}_{i}\cdot {\overrightarrow{r}}_{j}}{{\overrightarrow{r}}_{i}\cdot {\overrightarrow{r}}_{i}}=\frac{{\overrightarrow{s}}_{i}\cdot {\overrightarrow{s}}_{j}}{{\overrightarrow{s}}_{i}\cdot {\overrightarrow{s}}_{i}}=-\frac{1}{{N}^{2}-1},\,\forall i\ne j.$$

Equation (36) agrees with the requirement for pure state: angle *θ* between any two pure states must satisfy (see the Eq. (12) of ref.^37^)

37 $$-\,\frac{1}{N-1}\le \,\cos \,\theta \le 1.$$

As being true for qubit and numerically verified for qutrit systems, we are tempted to make the following conjecture:

#### **Conjecture 1**.

*There exists an* ${N}^{2}$-*simplex with circumradius* ${[\frac{\mathrm{2(}N-\mathrm{1)}}{N}]}^{\frac{1}{2}}$, *which fits into the convex hall of the* (${N}^{2}$ − 1)-*dimensional Bloch vector space of N*-*dimensional mixed states*.

### The separable decomposition for maximum value of $\frac{{\bf{2}}({\bf{N}}{\boldsymbol{\varphi }}-1)}{{\boldsymbol{N}}({{\boldsymbol{N}}}^{{\bf{2}}}-1)}$

Conjecture 1 leads to a solution to the open problem of finding separable decompositions of all separable Werner states in any dimension^38^. Inputting (35) to constraints for Bloch vectors, i.e. $|{\overrightarrow{r}}_{i}{|}^{2},|{\overrightarrow{s}}_{i}{|}^{2}\le \frac{\mathrm{2(}N-\mathrm{1)}}{N}$ (the equality holds for pure state), we have

38 $$|{\overrightarrow{r}}_{i}{|}^{2}={N}^{2}{\alpha }^{2}\,\sum _{j=1}^{{N}^{2}-1}\,{Q}_{ji}^{2}\le \frac{\mathrm{2(}N-\mathrm{1)}}{N},\,|{\overrightarrow{s}}_{i}{|}^{2}={N}^{2}{\beta }^{2}\,\sum _{j=1}^{{N}^{2}-1}\,{Q}_{ji}^{2}\le \frac{\mathrm{2(}N-\mathrm{1)}}{N}.$$

Because ${\sum }_{j=1}^{{N}^{2}-1}\,{Q}_{ji}^{2}=\frac{{N}^{2}-1}{{N}^{2}}$, Eq. (38) leads to

39 $${\alpha }^{2}\le \frac{2}{N(N+\mathrm{1)}},\,{\beta }^{2}\le \frac{2}{N(N+\mathrm{1)}}.$$

Inputting (33) and (34) into ${\mathscr{T}}={M}_{rp}{M}_{sp}^{{\rm{T}}}$ we have

40 $$\tau =|\frac{\mathrm{2(}N\varphi -\mathrm{1)}}{N({N}^{2}-\mathrm{1)}}|=\alpha \beta .$$

Combining of Eqs (39) and (40) leads to

41 $$|\frac{\mathrm{2(}N\varphi -\mathrm{1)}}{N({N}^{2}-\mathrm{1)}}|\le \frac{2}{N(N+\mathrm{1)}}\Rightarrow \frac{2}{N}-1\le \varphi \le 1.$$

The value of *ϕ* = 1 $({{\mathscr{T}}}_{ii}=\frac{2}{N(N+\mathrm{1)}})$ for *ρ*_*W*_ has the decomposition of two *N*^2^-simplexes in the *N*^2^ − 1 dimensional Bloch vector spaces of particles *A* and *B*, i.e.

42 $${\overrightarrow{r}}_{i}={\overrightarrow{s}}_{i}=\sqrt{\frac{2N}{N+1}}{({Q}_{1i},\ldots ,{Q}_{({N}^{2}-\mathrm{1)}i})}^{{\rm{T}}},\,i\in \{1,\ldots ,{N}^{2}\}.$$

### The separable decomposition for minimum value of $\frac{{\bf{2}}({\bf{N}}{\boldsymbol{\varphi }}-1)}{{\boldsymbol{N}}({{\boldsymbol{N}}}^{{\bf{2}}}-1)}$

If ${\mathscr{T}}$ is separable when *ϕ* = 1, and decomposes as

43 $${\mathscr{T}}=\frac{2}{N(N+\mathrm{1)}}1={M}_{rp}{M}_{rp}^{{\rm{T}}}$$

with ${M}_{rp}={M}_{sp}=\{\sqrt{{p}_{1}}\,{\overrightarrow{r}}_{1},\sqrt{{p}_{2}}\,{\overrightarrow{r}}_{2},\ldots \}$ and ${\overrightarrow{r}}_{i}$ being Bloch vectors for pure state, then for $\varphi =\frac{2}{N}-1$, ${\mathscr{T}}$ shall be written as

44 $${\mathscr{T}}=-\frac{2}{N(N+\mathrm{1)}}\mathbb{1}=-{M}_{rp}{M}_{rp}^{{\rm{T}}}={M}_{\overline{rp}}{M}_{rp}^{{\rm{T}}},$$

where ${M}_{\overline{rp}}=\{\sqrt{{p}_{1}}(-{\overrightarrow{r}}_{1}),\sqrt{{p}_{2}}(-{\overrightarrow{r}}_{2}),\ldots \}$. If ${\overrightarrow{r}}_{i}$ in Eq. (43) are Bloch vectors of pure state, $-{\overrightarrow{r}}_{i}$ in Eq. (44) can not be physical Bloch vectors for pure state according to Eq. (37)

45 $$\frac{(-{\overrightarrow{r}}_{i})\cdot {\overrightarrow{r}}_{i}}{|{\overrightarrow{r}}_{i}{|}^{2}}=-1\ngeqq -\frac{1}{N-1},$$

except for the qubit case of *N* = 2, where Bloch vectors of density matrix form a three dimensional ball. Therefore, the lower limit of *ϕ* is not $\frac{2}{N}-1$ except for the qubit case.

Now, suppose one of the two particles having Bloch vectors satisfying Observation 4, i.e. $|{\overrightarrow{r}}_{i}{|}^{2}\le \frac{2}{N(N-\mathrm{1)}}$ (or $|{\overrightarrow{s}}_{i}{|}^{2}\le \tfrac{2}{N(N-1)}$, but not both), by the procedure of Eqs (38) to (40), we have

46 $$\tau =|\frac{\mathrm{2(}N\varphi -\mathrm{1)}}{N({N}^{2}-\mathrm{1)}}|=\alpha \beta \le \frac{2}{N({N}^{2}-\mathrm{1)}}\Rightarrow 0\le \varphi \le \frac{1}{N}.$$

Therefore the separable decomposition for *ϕ* = 0 $({{\mathscr{T}}}_{ii}=-\frac{2}{N({N}^{2}-\mathrm{1)}})$ reads

47 $${\overrightarrow{r}}_{i}=-N\alpha {({Q}_{1i},{Q}_{2i},\ldots ,{Q}_{({N}^{2}-\mathrm{1)}i})}^{{\rm{T}}},\,{\overrightarrow{s}}_{i}=N\beta {({Q}_{1i},{Q}_{2i},\ldots ,{Q}_{({N}^{2}-\mathrm{1)}i})}^{{\rm{T}}},$$

where ${\alpha }^{2}=\frac{2}{N(N-\mathrm{1)}\,({N}^{2}-\mathrm{1)}}$ and ${\beta }^{2}=\frac{2}{N(N+\mathrm{1)}}$. The separable decomposition of Eq. (47) corresponds to two *N*^2^-simplexes: a smaller one composed with $\{{\overrightarrow{r}}_{i}\}$ and a larger one composed with $\{{\overrightarrow{s}}_{i}\}$. The smaller one satisfies reflection symmetry: because it lies in the Ball of $|\overrightarrow{r}{|}^{2}\le \frac{2}{N(N-\mathrm{1)}}$, both ${\overrightarrow{r}}_{i}$ and $-{\overrightarrow{r}}_{i}$ are Bloch vectors of density matrices.

## Discussion

We have presented an applicable and operational necessary and sufficient criterion for the separability of bipartite mixed state. The criterion is exhibited in a finite set of inequalities relating the correlation matrix to the Bloch vectors of the quantum states of subsystems, which is shown to be complete by exploring the multiplicative Horn’s problem. These inequalities may be treated as trade-off relations between the quantumness of the constituent parts, balanced by the correlation matrix. A state is separable if the decomposition can be performed within the convex hulls of the Bloch vectors of subsystems. As an illustration, separable decompositions for generalized Werner state and isotropic state are achieved in according to the new scheme. The proposed criterion sets up a geometric boundary in between the separability and entanglement for compound system, and provides a new perspective on the nonlocal nature of entanglement.

## Electronic supplementary material

Supplemental Material
