# Supplementary material for: A Necessary and Sufficient Criterion for the Separability of Quantum State
Source: Sci Rep. 2018 Jan 23;8:1442. doi: 10.1038/s41598-018-19709-z (PMC5780502; doi:10.1038/s41598-018-19709-z)
Supplement: Supplementary file 1 — Supplemental Material [file 41598_2018_19709_MOESM1_ESM.pdf]

A Necessary and Sufficient Criterion for the Separability of Quantum State
Supplemental Material

Jun-Li Li[1,3] and Cong-Feng Qiao[1,2,3*]

[1]Department of Physics, University of the Chinese Academy of Sciences,
YuQuan Road 19A, Beijing 100049, China

[2] Department of Physics & Astronomy, York University, Toronto, ON M3J 1P3, Canada

[3]Key Laboratory of Vacuum Physics, University of Chinese Academy of Sciences

[*] To whom correspondence should be addressed; E-mail: qiaocf@ucas.ac.cn.

Abstract

Quantum entanglement has been regarded as one of the key physical resources in quantum information sciences. However, the determination of whether a mixed state is entangled or not is generally a hard issue, even for the bipartite system. In this work we propose an operational necessary and sufficient criterion for the separability of an arbitrary bipartite mixed state, by virtue of the multiplicative Horn's problem. The work follows the work initiated by Horodecki *et. al.* and uses the Bloch vector representation introduced to the separability problem by J. De Vicente. In our criterion, a complete and finite set of inequalities to determine the separability of compound system is obtained, which may be viewed as trade-off relations between the quantumness of subsystems. We apply the obtained result to explicit examples, e.g. the separable decomposition of arbitrary dimension Werner state and isotropic state.

Supplemental Material

Here we present the detailed proofs and derivations for the theorems, observations, corollaries, and examples in the manuscript.

A Bloch representation of quantum states

A quantum state may be represented in form of Bloch vectors [S1]

$$\rho = \frac{1}{N}\mathbb{1} + \frac{1}{2}\sum_{\mu=1}^{N^2-1} r_\mu \lambda_\mu = \frac{1}{N}\mathbb{1} + \frac{1}{2}\vec{r}\cdot\vec{\lambda} \, , \tag{S1}$$

where the real coefficients $r_\mu = \langle \lambda_\mu \rangle = \mathrm{Tr}[\rho\lambda_\mu]$, and λ_μ are the N^2-1 traceless generators of $\mathrm{SU}(N)$ group. The N^2-1 dimensional real vectors $\vec{r}$ is the Bloch vector representation of the density matrix ρ. The normalization of the density matrices $\mathrm{Tr}[\rho^2] \leq 1$ imposes $|\vec{r}| \leq \sqrt{2(N-1)/N}$ where the vector norm is defined as $|\vec{r}| \equiv \sqrt{\vec{r}\cdot\vec{r}}$. The norm may be regarded as the mixedness (or quantumness) of the quantum state, as $|\vec{r}| = 0$ corresponds to the completely mixed states while $|\vec{r}| = \sqrt{2(N-1)/N}$ corresponds to the pure states. To ensure the positivity of the density operators, there are further constraints on the Bloch vectors $\vec{r}$ [S2, S3]. The whole space of the Bloch vectors $\vec{r}$ for quantum states (i.e., the density matrices satisfy the positivity and normalization conditions) forms convex hall in N^2-1 dimensional real space, whose circumscribed sphere and inscribed sphere have the radii of [S4]

$$R_+ = \sqrt{\frac{2(N-1)}{N}} \, , \; R_- = \sqrt{\frac{2}{N(N-1)}} \, , \tag{S2}$$

repectively.

An arbitrary bipartite state in the Bloch vector form is

$$\rho_{AB} \;=\; \frac{1}{NM}\mathbb{1}\otimes\mathbb{1} + \frac{1}{2M}\vec{a}\cdot\vec{\lambda}\otimes\mathbb{1} + \frac{1}{2N}\mathbb{1}\otimes\vec{b}\cdot\vec{\sigma} + \frac{1}{4}\sum_{\mu=1}^{N^2-1}\sum_{\nu=1}^{M^2-1}\mathcal{T}_{\mu\nu}\lambda_\mu\otimes\sigma_\nu \, , \tag{S3}$$

where $a_\mu = \mathrm{Tr}[\rho_{AB}(\lambda_\mu \otimes \mathbb{1})]$, $b_\nu = \mathrm{Tr}[\rho_{AB}(\mathbb{1} \otimes \sigma_\nu)]$, the correlation matrix $\mathcal{T}_{\mu\nu} = \mathrm{Tr}[\rho_{AB}(\lambda_\mu \otimes \lambda_\nu)]$, σ_ν are the generators of $\mathrm{SU}(M)$ and A and B are N and M dimensional subsystems. It could be transformed into the normal form

$$\rho_{AB} \to \widetilde{\rho}_{AB} = \frac{1}{NM}\mathbb{1} \otimes \mathbb{1} + \frac{1}{4}\sum_{\mu,\nu} \widetilde{\mathcal{T}}_{\mu\nu} \lambda_\mu \otimes \sigma_\nu \ , \tag{S4}$$

where the normal form has the same separability as the original state. Hereafter all the quantum state ρ_{AB} we considered are assumed to be in their normal form, i.e.

$$\rho_{AB} = \frac{1}{NM}\mathbb{1} \otimes \mathbb{1} + \frac{1}{4}\sum_{\mu,\nu} \mathcal{T}_{\mu\nu} \lambda_\mu \otimes \sigma_\nu \ . \tag{S5}$$

And we have the following observation

Observation S1 *Let $\vec{r}_i$ and $\vec{s}_j$ be Bloch vectors of density matrices and $\vec{p} = (p_1, p_2, \cdots, p_L)^{\mathrm{T}}$, we may define two matrices $M_{rp} \equiv M_r D_p^{\frac{1}{2}}$ and $M_{sp} \equiv M_s D_p^{\frac{1}{2}}$, where $M_r = \{\vec{r}_1, \vec{r}_2, \cdots, \vec{r}_L\}$, $M_s = \{\vec{s}_1, \vec{s}_2, \cdots, \vec{s}_L\}$, and $D_p = \mathrm{diag}\{p_1, p_2, \cdots, p_L\}$ with $0 < p_i \leq 1$, $\sum_{i=1}^{L} p_i = 1$. The state ρ_{AB} is separable if and only if there exist a number L such that $\mathcal{T} = M_{rp} M_{sp}^{\mathrm{T}}$ with $M_r \vec{p} = 0$ and $M_s \vec{p} = 0$.*

B The multiplicative Horn's problem for real square matrices

Let $\mathbb{R}^{n\downarrow}$, $\mathbb{R}_+^{n\downarrow}$, and $\mathbb{R}_+^{*n\downarrow}$ denote the sets of non-increasing sequences of real numbers, nonnegative real numbers, and strictly positive real numbers respectively. A set $\alpha = \{\alpha_1, \cdots, \alpha_n\} \in \mathbb{R}^{n\downarrow}$ means that $\forall i \in \{1, \cdots, n\}$, $\alpha_i \geq \alpha_{i+1}$ and $\alpha_i \in \mathbb{R}$. $\alpha \leq \beta$ for two sets $\alpha, \beta \in \mathbb{R}^{n\downarrow}$ means that $\forall i \in \{1, \cdots, n\}$, $\alpha_i \leq \beta_i$. $M_n(\mathbb{R})$ and $M_{m\times n}(\mathbb{R})$ denote the sets of real matrices of dimensions n by n and m by n respectively.

Theorem S1 *$\alpha, \beta, \gamma \in \mathbb{R}_+^{n\downarrow}$ occurs as singular values of n by n real matrices A, B, and*

$C = AB$ if and only if

$$\prod_{k\in K}\gamma_k \leq \prod_{i\in I}\alpha_i \cdot \prod_{j\in J}\beta_j \qquad \text{(S6)}$$

for all $(I, J, K) \in \bigcup_{r=1}^{n-1} T_r^n$.

We prove this theorem using the method of Ref. [S5]. To proceed the proof we first present the following 7 Lemmas.

Lemma S1 *$\alpha, \beta, \gamma \in \mathbb{R}^{n\downarrow}$ occurs as eigenvalues of Hermitian n by n matrices A, B, and C with $C = A + B$ if and only if*

$$\sum_{i=1}^{n}\gamma_i = \sum_{i=1}^{n}\alpha_i + \sum_{i=1}^{n}\beta_i \ , \qquad \text{(S7)}$$

$$\sum_{k\in K}\gamma_k \leq \sum_{i\in I}\alpha_i + \sum_{j\in J}\beta_j \ , \qquad \text{(S8)}$$

hold for every $(I, J, K) \in \bigcup_{r=1}^{n-1} T_r^n$.

This is the Horn's conjecture [S6] and has been proved in [S7, S8], see [S9] for a review on this subject where a systematical definition of T_r^n may also be found.

Lemma S2 *Let α_i, β_i, and γ_i be given real numbers, $1 \leq i \leq n$. The following conditions are equivalent: (1) there exist Hermitian matrices A, B, and C with eigenvalues α_i, β_i, and γ_i and sum $C = A+B$; (2) there exist real matrices X, Y, and Z with singular values e^{α_i}, e^{β_i}, and e^{γ_i} and product $XY = Z$.*

This is a direct corollary of Theorem 4.2 in [S10].

Lemma S3 *For sequences $\lambda = (\lambda_1, \cdots, \lambda_n)$, $\mu = (\mu_1, \cdots, \mu_n)$, $\nu = (\nu_1, \cdots, \nu_n)$ in $\mathbb{R}_{+}^{*n\downarrow}$, there exist matrices A, B, $C \in M_n(\mathbb{R})$ such that $C = AB$ and having singular values of*

λ, μ, and ν, respectively, if and only if

$$\prod_{i=1}^{n} \nu_i = \prod_{i=1}^{n} \lambda_i \mu_i \tag{S9}$$

$$\prod_{k\in K} \nu_k \le \prod_{i\in I} \lambda_i \prod_{j\in J} \mu_j \ . \tag{S10}$$

hold for all $(\lambda, \mu, \nu) \in \bigcup_{r=1}^{n-1} T_r^n$.

Proof: From Lemma S1, we have that the condition 1 of Lemma S2 is equivalent to equations (S7,S8) which may be reexpressed as

$$\exp(\sum_{i=1}^{n} \gamma_i) \ = \ \exp(\sum_{i=1}^{n} \alpha_i + \sum_{i=1}^{n} \beta_i) \ , \tag{S11}$$

$$\exp(\sum_{k\in K} \gamma_k) \ \le \ \exp(\sum_{i\in I} \alpha_i + \sum_{j\in J} \beta_j) \tag{S12}$$

hold for every $(I, J, K) \in \bigcup_{r=1}^{n-1} T_r^n$, where α, β, γ in $\mathbb{R}^{n\downarrow}$are eigenvalues of three Hermitian matrices with the third the sum of the first two. Equivalently, we have

$$\prod_{i=1}^{n} e^{\gamma_i} = \prod_{i=1}^{n} e^{\alpha_i} e^{\beta_i} \tag{S13}$$

$$\prod_{k\in K} e^{\gamma_k} \le \prod_{i\in I} e^{\alpha_i} \prod_{j\in J} e^{\beta_j} \tag{S14}$$

hold for every $(I, J, K) \in \bigcup_{r=1}^{n-1} T_r^n$. According to Lemma S2 the condition 2 is equivalent to condition 1 and therefore equivalent to

$$\prod_{i=1}^{n} \nu_i = \prod_{i=1}^{n} \lambda_i \mu_i \tag{S15}$$

$$\prod_{k\in K} \nu_k \le \prod_{i\in I} \lambda_i \prod_{j\in J} \mu_j \tag{S16}$$

hold for all $(I, J, K) \in \bigcup_{r=1}^{n-1} T_r^n$, where $\lambda_i = e^{\alpha_i}$, $\mu_i = e^{\beta_i}$, and $\nu_i = e^{\gamma_i}$. Q.E.D.

For $\lambda, \mu \in \mathbb{R}_+^{n\downarrow}$, define two sets $K_{\lambda,\mu}$ and $\widetilde{K}_{\lambda,\mu}$, where

$$K_{\lambda,\mu} \equiv \left\{ \nu \in \mathbb{R}_+^{n\downarrow} \mid \nu = \text{Singular values of diag}\{\lambda\}\ U\ \text{diag}\{\mu\},\ U \in O(n) \right\} \ , \tag{S17}$$

$$\widetilde{K}_{\lambda,\mu} \equiv \left\{ \nu \in \mathbb{R}_+^{n\downarrow} \mid \forall (I, J, K) \in \bigcup_{r=1}^{n-1} T_r^n, \prod_{i=1}^{n} \nu_i = \prod_{i=1}^{n} \lambda_i \mu_i, \prod_{k\in K} \nu_k \le \prod_{i\in I} \lambda_i \prod_{j\in J} \mu_j \right\} \ . \tag{S18}$$

Lemma S4 *If* $\lambda, \mu \in \mathbb{R}_+^{*n\downarrow}$*, then* $K_{\lambda,\mu} = \widetilde{K}_{\lambda,\mu}$.

Proof: This is just a different formulation of Lemma S3.

First, if $\nu \in K_{\lambda,\mu}$ then ν are the singular values of a matrix $C = AB$ where A, B have the singular values of λ, μ. According to Lemma S3, $\nu \in \widetilde{K}_{\lambda,\mu}$, that is $K_{\lambda,\mu} \subseteq \widetilde{K}_{\lambda,\mu}$.

Second, if $\nu \in \widetilde{K}_{\lambda,\mu}$ where $\lambda, \mu \in \mathbb{R}_+^{*n\downarrow}$, then according to Lemma S3 there exist real matrices $C = AB$ where A, B are real matrices and have the singular values of λ, μ. The singular value decomposition $A = U_a \mathrm{diag}\{\lambda\} V_a^{\mathrm{T}}$, $B = U_b \mathrm{diag}\{\lambda\} V_b^{\mathrm{T}}$, where $U_{a,b}, V_{a,b}$ are real orthogonal matrices, tells that $\nu \in K_{\lambda,\mu}$ and thus $\widetilde{K}_{\lambda,\mu} \subseteq K_{\lambda,\mu}$.

Therefore, we have $K_{\lambda,\mu} = \widetilde{K}_{\lambda,\mu}$. Q.E.D.

Lemma S5 *Let* $A, B \in M_n(\mathbb{R})$ *and let* $C = AB$*, and the singular values denote as* $\{\sigma_i\} \in \mathbb{R}_+^{n\downarrow}$*. Then for every* $(I, J, K) \in \bigcup_{r=0}^{n} T_r^n$ *the inequalities*

$$\sum_{k \in K} \log \sigma_k(C) \leq \sum_{i \in I} \log \sigma_i(A) + \sum_{j \in J} \log \sigma_j(B) \tag{S19}$$

holds, with $-\infty$ *allowed for the values of* log.

Proof: (See Theorem 3.5 of [S5]) Apply polar decomposition to A and B we have $A = U(\sqrt{A^\dagger A})$ and $B = (\sqrt{BB^\dagger})V$ where U, V are real orthogonal matrices. Since $C = U(\sqrt{A^\dagger A})(\sqrt{BB^\dagger})V$, we get that $\sigma_i(A) = \sigma_i(\sqrt{A^\dagger A})$, $\sigma_j(B) = \sigma_j(\sqrt{BB^\dagger})$, and $\sigma_k(C) = \sigma_k(\sqrt{A^\dagger A}\sqrt{BB^\dagger})$. Thus, without loss of generality we assume A and B are positive semidefinite. Let $\varepsilon_1, \varepsilon_2 > 0$ and let $C(\varepsilon_1, \varepsilon_2) = (A + \varepsilon_1 \mathbb{1})(B + \varepsilon_2 \mathbb{1})$, we have

$$\sum_{k \in K} \log[\sigma_k(C(\varepsilon_1, \varepsilon_2))] \leq \sum_{i \in I} \log[\varepsilon_1 + \sigma_i(A)] + \sum_{j \in J} \log[\varepsilon_2 + \sigma_j(B)] \ . \tag{S20}$$

By letting $\varepsilon_1, \varepsilon_2 \to 0$, we have $\sigma_k(C(\varepsilon_1, \varepsilon_2)) \to \sigma_k(C)$, and the right hand side of the inequality has possible values of $-\infty$. Q.E.D.

Define the complement of (I, J, K) in $\{1, \cdots, n\}$ as (I^c, J^c, K^c) where $I \cup I^c = \{1, \cdots, n\}$ and $I \cap I^c = \emptyset$ and similarly for J^c and K^c.

Lemma S6 *Let $\alpha',\alpha'',\beta',\beta'',\gamma',\gamma''\in\mathbb{R}^{n\downarrow}$, $\alpha'\le\alpha''$, $\beta'\le\beta''$, $\gamma'\le\gamma''$, and $\forall(I,J,K)\in\bigcup_{r=0}^{n}T_r^n$ the inequalities*

$$\begin{aligned}\sum_{k\in K}\gamma'_k &\le \sum_{i\in I}\alpha''_i+\sum_{j\in J}\beta''_j\ , &\text{(S21)}\\ \sum_{k\in K^c}\gamma''_k &\ge \sum_{i\in I^c}\alpha'_i+\sum_{j\in J^c}\beta'_j\ , &\text{(S22)}\end{aligned}$$

hold, then there exist $\alpha'\le\alpha\le\alpha''$, $\beta'\le\beta\le\beta''$, $\gamma'\le\gamma\le\gamma''$ that

$$\begin{aligned}\sum_{i=1}^{n}\gamma_i &= \sum_{i=1}^{n}\alpha_i+\sum_{j=1}^{n}\beta_j\ , &\text{(S23)}\\ \sum_{k\in K}\gamma_k &\le \sum_{i\in I}\alpha_i+\sum_{j\in J}\beta_j\ . &\text{(S24)}\end{aligned}$$

hold for all $(I,J,K)\in\bigcup_{r=1}^{n-1}T_r^n$.

Lemma S6 comes from the Proposition 2.1 of [S5] and Proposition 3.2 of [S11].

Define two sets

$$\begin{aligned}\widetilde{K}^{\le}_{\lambda,\mu} &= \left\{\nu\in\mathbb{R}_+^{n\downarrow}|\forall(I,J,K)\in\bigcup_{r=0}^{n}T_r^n,\ \prod_{k\in K}\nu_k\le\prod_{i\in I}\lambda_i\prod_{j\in J}\mu_j\right\}\ , &\text{(S25)}\\ \widetilde{K}^{\ge}_{\lambda,\mu} &= \left\{\nu\in\mathbb{R}_+^{n\downarrow}|\forall(I,J,K)\in\bigcup_{r=0}^{n}T_r^n,\ \prod_{k\in K^c}\nu_k\ge\prod_{i\in I^c}\lambda_i\prod_{j\in J^c}\mu_j\right\}\ . &\text{(S26)}\end{aligned}$$

Note that $\widetilde{K}_{\lambda,\mu}=\widetilde{K}^{\le}_{\lambda,\mu}\cap\widetilde{K}^{\ge}_{\lambda,\mu}$, that is the elements both belong to $\widetilde{K}^{\le}_{\lambda,\mu}$ and $\widetilde{K}^{\ge}_{\lambda,\mu}$ take up the whole set of $\widetilde{K}_{\lambda,\mu}$. Considering the case of $r=n$ where all $I,J,K=\{1,\cdots,n\}$, we have $\prod_{i=1}^n\nu_i\le\prod_{i=1}^n\lambda_i\prod_{i=1}^n\mu_i$ from $\widetilde{K}^{\le}_{\lambda,\mu}$. When $r=0$ we have $\prod_{i=1}^n\nu_i\ge\prod_{i=1}^n\lambda_i\prod_{i=1}^n\mu_i$ from $\widetilde{K}^{\ge}_{\lambda,\mu}$. So the values in $\widetilde{K}^{\le}_{\lambda,\mu}\cap\widetilde{K}^{\ge}_{\lambda,\mu}$ have $\prod_{i=1}^n\nu_i=\prod_{i=1}^n\lambda_i\prod_{i=1}^n\mu_i$.

Now we begin to proof Theorem S1. We need only prove the following lemma

Lemma S7 *For all $\lambda,\mu\in\mathbb{R}_+^{n\downarrow}$, we have $K_{\lambda,\mu}=\widetilde{K}_{\lambda,\mu}$.*

Proof: First, if $\nu\in K_{\lambda,\mu}$ from Lemma S5 we have $\nu\in\widetilde{K}_{\lambda,\mu}$ and $K_{\lambda,\mu}\subseteq\widetilde{K}_{\lambda,\mu}$.

Second, if $\nu \in \widetilde{K}_{\lambda,\mu}$, for an arbitrary small positive real number ε, it is clear that, $\nu+\varepsilon \in \widetilde{K}^{\geq}_{\lambda,\mu}$, similarly $\nu \in \widetilde{K}^{\leq}_{\lambda+\varepsilon,\mu+\varepsilon}$. There exist $\delta = \delta(\varepsilon)$ satisfying $0 < \delta < \varepsilon$ such that $\nu+\varepsilon \in \widetilde{K}^{\geq}_{\lambda+\delta,\mu+\delta}$ and $\nu+\delta \in \widetilde{K}^{\leq}_{\lambda+\varepsilon,\mu+\varepsilon}$. Thus there exist ν_k that both of the following two groups of inequalities

$$\sum_{k\in K} \log(\nu_k+\delta) \leq \sum_{i\in I} \log(\lambda_i+\varepsilon) + \sum_{j\in J} \log(\mu_j+\varepsilon) \tag{S27}$$

$$\sum_{k\in K^c} \log(\nu_k+\varepsilon) \geq \sum_{i\in I^c} \log(\lambda_i+\delta) + \sum_{j\in J^c} \log(\mu_j+\delta) \tag{S28}$$

hold for $(I,J,K) \in \bigcup_{r=0}^{n} T_r^n$. Thus according to Lemma S6 we have

$$\log(\lambda+\delta) \leq \alpha \leq \log(\lambda+\varepsilon) \tag{S29}$$

$$\log(\mu+\delta) \leq \beta \leq \log(\mu+\varepsilon) \tag{S30}$$

$$\log(\nu+\delta) \leq \gamma \leq \log(\nu+\varepsilon) \tag{S31}$$

where $\sum_{i=1}^{n} \gamma_i = \sum_{i=1}^{n} \alpha_i + \sum_{i=1}^{n} \beta_i$ and $\sum_{k\in K} \gamma_k \leq \sum_{i\in I} \alpha_i + \sum_{j\in J} \beta_j$ for all $(I,J,K) \in \bigcup_{r=1}^{n-1} T_r^n$. Here the inequalities are all assumed to be established componentwise following the definition in the beginning of Sec. B. Letting $\lambda_\varepsilon = e^{\alpha}$, $\mu_\varepsilon = e^{\beta}$, and $\nu_\varepsilon = e^{\gamma}$, we have

$$\lambda+\delta(\varepsilon) \leq \lambda_\varepsilon \leq \lambda+\varepsilon \ , \ \mu+\delta(\varepsilon) \leq \mu_\varepsilon \leq \mu+\varepsilon \ , \ \nu+\delta(\varepsilon) \leq \nu_\varepsilon \leq \nu+\varepsilon \ . \tag{S32}$$

As $\nu_\varepsilon \in \widetilde{K}_{\lambda_\varepsilon,\mu_\varepsilon}$, Lemma S4 gives that $\nu_\varepsilon \in K_{\lambda_\varepsilon,\mu_\varepsilon}$, so there is an real orthogonal matrix U_ε so that the singular values of $\mathrm{diag}(\lambda_\varepsilon)U_\varepsilon\mathrm{diag}(\mu_\varepsilon)$ are precisely ν_ε. Choosing a sequence $\varepsilon(k)$ tending to zero as $k \to \infty$, so that $U_{\varepsilon(k)}$ converge to a real orthogonal matrix U as $k \to \infty$. We have that the singular values of $\mathrm{diag}(\lambda)U\mathrm{diag}(\mu)$ are precisely ν based on Eq. (S32). Thus $\nu \in K_{\lambda,\mu}$ and $\widetilde{K}_{\lambda,\mu} \subseteq K_{\lambda,\mu}$.

From the above discussions we have $K_{\lambda,\mu} = \widetilde{K}_{\lambda,\mu}$. Q.E.D.

The Lemma S7 is equivalent to Theorem S1.

C Examples

C.1 Comparing with the PPT criterion

The partial transposition is an operation on the density matrix that makes the transposition only on one of the composed particles, i.e.,

$$(A \otimes B)^{\mathrm{T_B}} = A \otimes B^{\mathrm{T}} \ . \tag{S33}$$

So a partial transposition of a separable state

$$\rho_{AB} = \sum_k p_k \rho_k^{(A)} \otimes \rho_k^{(B)} \ , \ \sum_k p_k = 1 \ , \ p_k > 0 \tag{S34}$$

takes the form of

$$\rho'_{AB} = \rho_{AB}^{\mathrm{T_B}} = \sum_k p_k \rho_k^{(A)} \otimes \rho_k'^{(B)} \ , \ \rho_k'^{(B)} = \rho_k^{(B)\mathrm{T}} \ , \tag{S35}$$

which is also a separable quantum state and thus positive definite. Therefore, the positive partial transposition criterion is a necessary condition for the quantum state to be separable: if a quantum state is separable then the quantum state and its partial transposition are positive definite.

In our method, the partial transposition on partite B of quantum state ρ_{AB} is

$$\begin{aligned} (\rho_{AB})^{\mathrm{T_B}} &= \frac{1}{N^2}\mathbb{1} \otimes \mathbb{1} + \frac{1}{4}\sum_{\mu,\nu=1}^{N^2-1} t_{\mu\nu}\lambda_\mu \otimes \lambda_\nu^{\mathrm{T}} \\ &= \frac{1}{N^2}\mathbb{1} \otimes \mathbb{1} + \frac{1}{4}\sum_{\mu,\nu=1}^{N^2-1} t'_{\mu\nu}\lambda_\mu \otimes \lambda_\nu \ , \end{aligned} \tag{S36}$$

where $t'_{\mu\nu} = -t_{\mu\nu}$ for the columns that $\lambda_\nu^{\mathrm{T}} = -\lambda_\nu$. If ρ_{AB} is separable that $\mathcal{T} = M_r M_s^{\mathrm{T}}$, then

$$(\rho_{AB})^{\mathrm{T_B}} = \frac{1}{N^2}\mathbb{1} \otimes \mathbb{1} + \frac{1}{4}\sum_{k=1}^{n} p_k \vec{r}_k \cdot \vec{\lambda} \otimes \vec{s}_k{}' \cdot \vec{\lambda} \ , \tag{S37}$$

where $\vec{r}_k \cdot \vec{\lambda} = \sum_\mu (r_k)_\mu \lambda_\mu$, $\vec{s}_k{}' \cdot \vec{\lambda} = \sum_\mu (s'_k)_\nu \lambda_\nu$, and $(s'_k)_\nu = -(s_k)_\nu$ for the skew symmetric λ_ν. Because the transposition of the density matrix of one particle quantum state is also a density matrix of one particle quantum state, so $(\rho_{AB})^{\mathrm{T_B}}$ is also a separable state that is positive definite, i.e., PPT is necessary.

The sufficient part of the PPT criterion is: if $(\rho_{AB})^{\mathrm{T_B}}$ is positive definite (ρ_{AB} is positive definite by definition) then ρ_{AB} is separable. This statement is not true for general bipartite states. Here we show that it is true for qubit-qubit systems. In the normal forms, the two-qubit quantum state may be represented as $\rho_{AB} = \frac{1}{4} x_{\mu\nu} D_{\mu\nu}$ [S12], where $D_{\mu\nu} = \sigma_\mu \otimes \sigma_\nu$, $\sigma_0 = \mathbb{1}$, and $\sigma_{1,2,3}$ are Pauli matrices. The relation of $X = x_{\mu\nu}$ ($\mu, \nu \in \{0, 1, 2, 3\}$) with $\mathcal{T} = t_{\mu\nu}$ ($\mu, \nu \in \{1, 2, 3\}$) is

$$X = \begin{pmatrix} 1 & 0 & 0 & 0 \\ 0 & t_{11} & t_{12} & t_{13} \\ 0 & t_{21} & t_{22} & t_{23} \\ 0 & t_{13} & t_{23} & t_{33} \end{pmatrix} = \begin{pmatrix} 1 & 0 \\ 0 & \mathcal{T} \end{pmatrix} . \tag{S38}$$

The positivity condition of ρ_{AB} requires (see Eq. (54) in [S12])

$$4 - ||X||^2 \geq 0 , \tag{S39}$$

$$-2\mathrm{det}\mathcal{T} - (||X||^2 - 2) \geq 0 , \tag{S40}$$

$$-8\mathrm{det}\mathcal{T} + (||X||^2 - 2)^2 - 4(\tau_2^2\tau_3^2 + \tau_3^2\tau_1^2 + \tau_1^2\tau_2^2) \geq 0 . \tag{S41}$$

where $||X||^2 = \mathrm{Tr}[X^\dagger X]$, τ_i are the singular values of $\mathcal{T}$. After the partial transposition, $\rho_{AB}^{\mathrm{T_B}}$ has the X' in following form

$$X' = \begin{pmatrix} 1 & 0 & 0 & 0 \\ 0 & t_{11} & -t_{12} & t_{13} \\ 0 & t_{21} & -t_{22} & t_{23} \\ 0 & t_{13} & -t_{23} & t_{33} \end{pmatrix} , \ \mathcal{T}' = \begin{pmatrix} t_{11} & -t_{12} & t_{13} \\ t_{21} & -t_{22} & t_{23} \\ t_{13} & -t_{23} & t_{33} \end{pmatrix} . \tag{S42}$$

Therefore the positivity condition of $\rho_{AB}^{\mathrm{T_B}}$ requires

$$4 - ||X||^2 \geq 0 \ , \tag{S43}$$

$$2\det\mathcal{T} - (||X||^2 - 2) \geq 0 \ , \tag{S44}$$

$$8\det\mathcal{T} + (||X||^2 - 2)^2 - 4(\tau_2^2\tau_3^2 + \tau_3^2\tau_1^2 + \tau_1^2\tau_2^2) \geq 0 \ , \tag{S45}$$

where we have used the fact $\det[\mathcal{T}'] = -\det[\mathcal{T}]$ and the singular values of $\mathcal{T}'$ and $\mathcal{T}$ are the same. The two group of positivity inequalities should be satisfied simultaneously, so we have

$$\tau_1^2 + \tau_2^2 + \tau_3^2 \leq 3 \ , \tag{S46}$$

$$\pm 2\tau_1\tau_2\tau_3 - (\tau_1^2 + \tau_2^2 + \tau_3^2 - 1) \geq 0 \ , \tag{S47}$$

$$\pm 8\tau_1\tau_2\tau_3 + (\tau_1^2 + \tau_2^2 + \tau_3^2 - 1)^2 - 4(\tau_2^2\tau_3^2 + \tau_3^2\tau_1^2 + \tau_1^2\tau_2^2) \geq 0 \ , \tag{S48}$$

which be reduced to

$$\tau_1^2 + \tau_2^2 + \tau_3^2 \leq 3 \ , \tag{S49}$$

$$\tau_1^2 + \tau_2^2 + \tau_3^2 + 2\tau_1\tau_2\tau_3 \leq 1 \ , \tag{S50}$$

$$(\tau_1 - \tau_2 - \tau_3 - 1)(\tau_1 + \tau_2 - \tau_3 + 1)(\tau_1 - \tau_2 + \tau_3 + 1)(\tau_1 + \tau_2 + \tau_3 - 1) \geq 0 \ . \tag{S51}$$

Further reductions shows that $0 \leq \tau_1 + \tau_2 + \tau_3 \leq 1$, and according Corollary 2 we have the qubit-qubit system is separable. Therefore the PPT critrion is necessary and sufficient for qubit-qubit systems.

C.2 The decomposition of the generalized Werner state

Here we present a numerical result for the decomposition of qutrit Werner state

$$\rho_{\mathrm{W}} = \frac{1}{9}\mathbb{1} \otimes \mathbb{1} + \frac{1}{4}\sum_{\mu=1}^{8} \frac{2(3\phi - 1)}{3 \times 8}\lambda_\mu \otimes \lambda_\mu \ . \tag{S52}$$

For $\phi = 1$ we have

$$\rho_{\rm W} = \frac{1}{9}\mathbb{1}\otimes\mathbb{1} + \frac{1}{4}\sum_{\mu=1}^{8}\frac{1}{6}\lambda_\mu\otimes\lambda_\mu \ . \tag{S53}$$

The Bloch vectors are

$$\vec{r}_i = (\frac{3}{2})^{\frac{1}{2}}(Q_{1i}, Q_{2i}, \cdots, Q_{8i})^{\rm T} \ , \tag{S54}$$

$$\vec{s}_i = (\frac{3}{2})^{\frac{1}{2}}(Q_{1i}, Q_{2i}, \cdots, Q_{8i})^{\rm T} \ , \tag{S55}$$

where $i \in \{1, \cdots, 9\}$. The constraints of Q_{ij} ($Q_{9i} = \frac{1}{3}$) are

$$\sum_{k=1}^{8} Q_{ki}Q_{kj} = \begin{cases} \frac{8}{9} & i = j \\ \\ \frac{-1}{9} & i \neq j \end{cases} \ , \tag{S56}$$

$$-\frac{4}{9} + \frac{1}{2}\left(\frac{3}{2}\right)^{\frac{3}{2}} d_{\mu\nu\rho}Q_{\mu i}Q_{\nu i}Q_{\rho i} = 0 \ , \tag{S57}$$

where Eq. (S57) ensures that the density matrices of $\rho_i^{(A)}$ and $\rho_i^{(B)}$ are all positive semidefinite [S3]. Numerical analysis of Eqs. (S56, S57) could be evaluated using math softwares (there exist subroutines in Mathematica or MATLAB).

C.3 Pure Bell state

For Bell state $|\psi\rangle_{AB} = \frac{1}{\sqrt{2}}(|00\rangle + |11\rangle)$, its density matrix is

$$\rho_{AB} = \frac{1}{2}\begin{pmatrix} 1 & 0 & 0 & 1 \\ 0 & 0 & 0 & 0 \\ 0 & 0 & 0 & 0 \\ 1 & 0 & 0 & 1 \end{pmatrix} = \frac{1}{4}(\mathbb{1}\otimes\mathbb{1} + \sigma_x\otimes\sigma_x - \sigma_y\otimes\sigma_y + \sigma_z\otimes\sigma_z) \ . \tag{S58}$$

The correlation matrix $\mathcal{T} = \mathrm{diag}\{1, -1, 1\}$ and has the singular values $\tau_1 = \tau_2 = \tau_3 = 1$. Corollary 1 leads to $(\tau_1 + \tau_2 + \tau_3)^2 = (\sum_i p_i|\vec{r}_i|^2)(\sum_j p_j|\vec{s}_j|^2)$, where no Bloch vectors $\vec{r}_i$ and $\vec{s}_j$ of qubit systems satisfy the relation ($\tau_1 + \tau_2 + \tau_3 = 3$ while $\sum_i p_i = 1$, $|\vec{r}_i|^2 \leq 1$ and $|\vec{s}_i|^2 \leq 1$).

C.4 P-zero state

Consider the following bipartite qubit state [S4]

$$\rho_\pm = p|\psi^\pm\rangle\langle\psi^\pm| + (1-p)|00\rangle\langle 00| \,, \tag{S59}$$

where $0 \le p \le 1$ and $|\psi^\pm\rangle = \frac{1}{\sqrt{2}}(|01\rangle \pm |10\rangle)$. If $p = 0$ then $\rho_\pm$ is a separable state. If $p \neq 0$, we may apply the procedure of [S13] to transform $\rho_\pm$ to their normal forms. For the case of ρ_+, after N steps of transformations, we have

$$\widetilde{\rho}_+ = \frac{1}{2}\begin{pmatrix} \frac{2-2p}{N+1-Np} & 0 & 0 & 0 \\ 0 & \frac{N-1-(N-2)p}{N+1-Np} & \sqrt{\frac{N-1-(N-2)p}{N+1-Np}} & 0 \\ 0 & \sqrt{\frac{N-1-(N-2)p}{N+1-Np}} & 1 & 0 \\ 0 & 0 & 0 & 0 \end{pmatrix} . \tag{S60}$$

We see that its normal form is $\lim_{N\to\infty} \widetilde{\rho}_+ = |\psi\rangle\langle\psi|$, where $|\psi\rangle = \frac{1}{\sqrt{2}}(|01\rangle + |10\rangle)$. Similar process applies to ρ_-, therefore $\rho_\pm$ is separable only when $p = 0$.

References

[S1] F. T. Hioe and J. H. Eberly, N-Level coherence vector and higher conservation laws in quantum optics and quantum mechanics, Phys. Rev. Lett. **47,** 838-841 (1981).

[S2] M. S. Byrd and N. Khaneja, Characterization of the positivity of the density matrix in terms of the coherence vector representation, Phys. Rev. A **68,** 062322 (2003).

[S3] G. Kimura, The Bloch vector for N-level systems, Phys. Lett. A **314,** 339-349 (2003).

[S4] J. I. de Vicente, Separability criteria based on the Bloch representation of density matrices, Quantum Inf. Comput. **7,** 624-638 (2007).

[S5] H. Bercovici, B. Collins, K. Dykema, and W. S. Li, Characterization of singular numbers of products of operators in matrix algebras and finite von Neumann algebras, Bull. Sci. Math. **139,** 400-419 (2015).

[S6] A. Horn, Eigenvalues of sums of Hermitian matrices, Pacific J. Math. **12**, 225-241 (1962).

[S7] A. A. Klyachko, Stable bundles, representation theory and Hermitian operators, Selecta Math. **4,** 419-445 (1998).

[S8] A. Knutson and T. Tao, The honeycomb model of $\mathrm{GL}_n(\mathbb{C})$ tensor products I: Proof of the saturation conjecture, J. Amer. Math. Soc. **12,** 1055-1090 (1999).

[S9] W. Fulton, Eigenvalues, invariant factors, highest weights, and Schubert calculus, Bull. Ame. Math. Soc. **37,** 209-249 (2000).

[S10] A. Alekseev, E. Meinrenken, and C. Woodward, Linearization of Poisson actions and singular values of matrix products, Ann. Inst. Fourier **51,** 1691-1717 (2001).

[S11] H. Bercovici, W. S. Li, and D. Timotin, The Horn conjecture for sums of compact selfadjoint operators, Am. J. Math. **131,** 1543-1567 (2009).

[S12] O. Gamel, Entangled Bloch spheres: Bloch matrix and two-qubit state space, Phys. Rev. A **93,** 062320 (2016).

[S13] F. Verstraete, J. Dehaene, and B. De Moor, Normal forms and entanglement measures for multipartite quantum states, Phys. Rev. A **68,** 012103 (2003).
